# Knowledge, Attitude, and Practice (KAP) of Dairy Products in Chinese Urban Population and the Effects on Dairy Intake Quality

**DOI:** 10.3390/nu9070668

**Published:** 2017-06-27

**Authors:** Ai Zhao, Ignatius Man-Yau Szeto, Yan Wang, Ce Li, Min Pan, Ting Li, Peiyu Wang, Yumei Zhang

**Affiliations:** 1 School of Public Health, Peking University Health Science Center, Beijing 100191, China; aizhao@bjmu.edu.cn (A.Z.); wpeiyu@bjmu.edu.cn (P.W.); 2 Inner Mongolia Dairy Technology Research Institute Co. Ltd., Hohhot 010110, China; szeto@yili.com (I.M.-Y.S.); wangyan0@yili.com (Y.W.) lice@yili.com (C.L.); panmin@yili.com (M.P.); liting2012@yili.com (T.L.); 3 Yili Innovation Center, Inner Mongolia Yili Industrial Group Co. Ltd., Hohhot 010110, China

**Keywords:** dairy, knowledge, attitude, practice, nutritional self-perception, diet quality, calcium

## Abstract

Insufficient intake of dairy products is a nutritional problem of concern in China. However, the knowledge, attitude, and practice of consuming dairy products in the Chinese population remain unknown. A total of 1739 subjects from eight cities in China participated in this study. A questionnaire was used to measure knowledge of and attitude toward dairy. A semi-food intake frequency questionnaire was used to obtain the frequencies and amount of different kinds of dairy product intake. Calcium and protein intake were investigated within one 24-hour period of dietary recall. The results showed that questions related to lactose intolerance had the highest unknown rate and the lowest accuracy. Males, subjects with a lower education level, lower monthly family income (<3000 yuan), lower monthly family food expenditure (<1000 yuan), and lower personal monthly expenditure on dairy products (<10 yuan) had a significantly lower knowledge score. For attitude, 42.7 percent of subjects had self-perceived insufficient intake of dairy. While 15.2 percent of subjects reported experiencing lactose intolerance symptoms, 29.5 percent did not know the reasons. In practice, the median (25th, 75th) intake of dairy products was 71.4 (0.0, 200.0) g/day. A significantly lower intake of dairy and low-fat or fat-free dairy products was shown in subjects with poor dairy knowledge. For the perception of lactose intolerance, the lowest intake was shown in the “unsure” group. In conclusion, knowledge gaps and self-perception bias regarding dairy products exist in Chinese urban adults and these are associated with the quantity and quality of dairy intake.

## 1. Introduction

In both western and Asian countries, dairy consumption is recommended as part of a healthy diet. Dietary Guidelines for Americans (DGA) 2015–2020 recommends fat-free or low fat dairy, including milk, yogurt, and cheese, as a key food in a healthy food pattern [1]. The updated Dietary Guidelines for Chinese Residents 2016 also recommends increased dairy intake of 300–500 g per day [2]. The explicit assumption is that adequate intake of dairy products is essential for extending life span, especially for promoting bone health [3]. In addition, although the study results are inconsistent, many studies have reported that an appropriate intake of dairy products could be of benefit by reducing the risk of many chronic diseases, such as cardiovascular disease and diabetes [4,5,6,7]. However, it is disappointing to realize that the public seems to be unmoved by this message [5]. From 1986 to 2011, the daily intake of dairy products by Chinese residents significantly increased in both urban (from 44.0 g to 74.0 g) and rural areas (from 0.13 g to 22.9 g), but it was still far lower than the recommended level [8]. This leads to one of the biggest nutritional concerns in China: the highly insufficient intake of calcium [9].

There is a paucity of information from China and other countries on the causes and/or associated factors of insufficient dairy intake. Lactose intolerance (LI) might be an important factor in the East Asian and African population [10]. The prevalence of insufficient lactase in these two populations was as high as 65–85 percent [11]. There are no available updated data on LI prevalence or the lactase deficiency rate in Chinese adults: one study in 1984 reported that 86.1 percent of studied subjects were lactose malabsorbers [12]; another study in 1999 reported that the incidence of lactase deficiency in Chinese children aged 3–5 years, 7–8 years, and 11–13 years was 38.5 percent, 87.6 percent, and 87.8 percent respectively, increasing with age [13]. However, as food technology has advanced, lactose-reduced dairy products and enzyme-replacement dairy products can both be flexible options for lactose-intolerant people [14].

Despite the above possible reasons, health-related behavior, based on behavioral theories, is affected by different aspects of knowledge, attitude, and practice (KAP). Nutritional awareness can greatly impact diet quality [15]. It is worth noting that an Iranian study indicated that a great nutritional knowledge gap exists in residents in respect to dairy [16]. Dairy knowledge in the Chinese population is still unknown. Several studies have also revealed that awareness might influence dairy product expenditure [5]. A nutritional behavior intervention study revealed that using a KAP model to promote nutritional attitude can significantly increase dairy product intake in Iran [17]. These findings triggered our interest in the following queries: (1) whether a nutritional knowledge gaps concerning dairy also exist in the Chinese population; and (2) whether improper knowledge and attitude contribute to the insufficient intake of dairy products in China.

## 2. Materials and Methods

### 2.1. Subjects

This study is part of the Chinese Urban Adults Diet and Health Study, which was a cross-sectional survey carried out in eight Chinese cities between March and July 2016. A multistage sampling method was used to recruit subjects. As a first step, we purposively selected eight cities in China according to geographical location and economic status: according to China’s city levels, Beijing and Guangzhou are categorized as first-tier cities, which have a higher economic level, and Xuchang, Jilin, Wuhu, Lanzhou, Chenzhou, and Chengdu are categorized as non–first tier cities. In a second step, in each first-tier city, two communities were selected by a convenience sampling method according to economic status, while one community was selected in each non–first tier city. Then, based on the resident registration, subjects were divided into three age groups: 18–44 years, 45–64 years, and >65 years. In each age group, at least 60, 60, and 50 residents were randomly selected respectively. A total of 1806 subjects were invited and, finally, a total of 1739 subjects were considered eligible and willing to participate in this study. Those with a physical disability, mental illness, or memory problems were excluded.

### 2.2. Data Collection

Data were collected from subjects by trained interviewers using an interviewer-administered questionnaire in two parts related to socio-demographic factors and KAP. For knowledge, a total of six questions were used to measure subjects’ dairy knowledge. The individual received one point for each correct answer, −1 point for a wrong answer, and 0 points if they said “don’t know.” Meanwhile, personal attitude toward dairy and LI and dairy-related practices were investigated. Training of the interviewers, an initial site survey, and preliminary questionnaire testing were completed prior to data collection.

For dairy intake, the Semi-quantitative Food Frequency Questionnaire (semi-FFQ) was used to investigate the intake of dairy products in one recent month. There are 41 items in this semi-FFQ, 21 items regarding the general food groups according to Dietary Guidelines for Chinese Residents 2016 [2], and another 20 items that were developed in this study to investigate different types of dairy product (such as whole-fat milk, low fat milk, skim milk, lactose-reduced milk, cheese, formula, etc.). One time 24-hour dietary recall was used to obtain the data on food intake and calculate the nutrients intake. Standard bowls, plates, and spoons and the standard reference picture book were used to help with quantification of food consumption. Protein and calcium were analyzed based on the Chinese Food Composition Table (second edition) and the nutrient composition table on the food packaging [18].

### 2.3. Ethics

The study was conducted in compliance with the Declaration of Helsinki. All of the procedures involving human subjects were approved by the Medical Ethics Research Board of Peking University (No. IRB00001052-15059). Written consent was obtained from the participants before the study began.

### 2.4. Statistical Analysis

IBM SPSS (predictive analytics software and solutions) version 20.0 (International Business Machines Corporation, Armonk, NY, USA) was used for analysis. Average food intakes were compared with the recommended daily food intake based on the Chinese balanced diet pagoda [19]. Normality was tested for all data before analysis. Values were presented as mean, median (25th, 75th), or percentage. Chi-squared analysis was used to compare socio-demographic characteristics among participants with different knowledge levels prior to the ordinal regression analysis and only significant factors were involved in the regression model. Ordinal logistic regression was used to explore the associations between socio-demographic indicators and dairy knowledge level and obtain the regression coefficients (ORs) and 95% CI. A nonparametric test was used to exam the differences in dairy intake among subjects with different knowledge levels and different self-perception of LI-related symptoms. The linear regression model was used to explore the associations between nutrients intake and dairy knowledge level. A *P* value less than 0.05 was considered as statistically significantly different.

## 3. Results

### 3.1. Socio-Demographic Characteristics of Participants

Out of 1739 recruited subjects, data from 1714 participants were used for analysis. Twenty-five subjects were excluded because of missing values in the key questions. The socio-demographic characteristics of participants are shown in Table 1.

### 3.2. Knowledge of Dairy among Participants

**Figure 1:**
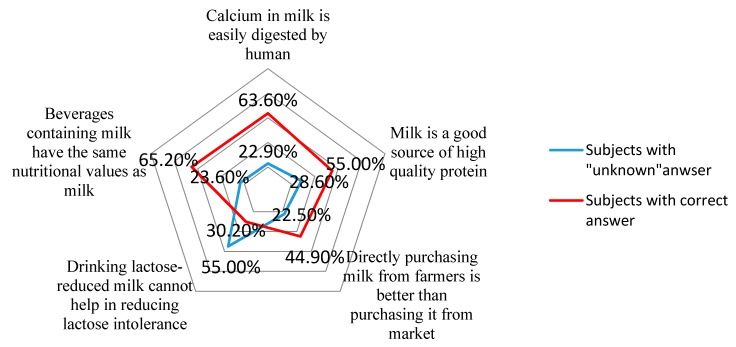
The dairy product knowledge of Chinese urban adults.

The answers to each question are shown in Figure 1. Among the six questions, over half of the subjects have a “don’t know” answer in response to lactose intolerance. Questions concerning LI had the highest unknown rate and the lowest accuracy. To calculate the total score of knowledge, the median (25th, 75th) point was 2 (0, 4). Of the subjects, 28.2 percent, 41.0 percent, and 30.8 percent scored −4 to 0 points, 1 to 3 points and ≥4 points respectively.

A lower education level, being male, family monthly income <3000 yuan, family monthly expenditure on food <1000 yuan, and average personal monthly expenditure on dairy products <100 yuan were associated with a lower knowledge level (see Table 2). Age levels and city levels were not associated with dairy knowledge.

### 3.3. Attitude toward Dairy Products among Participants

When investigating participants’ self-perception of potential dietary problems, the top three most frequently mentioned problems were: (1) insufficient intake of fruits and vegetables (42.9%); (2) insufficient intake of dairy products (42.7%); and (3) low dietary diversity (33.2%).

Of the 1714 subjects, 260 (15.2%) reported that they experienced a series of LI symptoms after consuming dairy products; 75.2 percent and 9.6 percent, respectively, perceived no LI-related symptoms or were unsure. In the subjects with symptoms, 29.5 percent did not know the reasons, 21.7 percent perceived that the symptoms might be related to LI, and the other 48.0 percent and 0.8 percent respectively thought the symptoms were caused by gastrointestinal problems and sour milk.

### 3.4. Diet Quality and Associated Predictors

Based on the data from the semi-FFQ, we estimated daily dairy products intake. The median (25th, 75th) intake of total dairy products was 71.4 (0.0, 200.0) g/day. Only 4.3 percent of participants’ daily intake of dairy products reached the recommended level (300 g/day). Among the participants who did not reach the recommended level, only 44.0 percent had correct self-perception of insufficient intake, while 56.0 percent did not. For different kinds of dairy product, only 13.1 percent, 32.1 percent, and 2.8 percent of participants had consumed low fat or fat-free dairy products, yogurt, and lactose-reduced dairy products respectively once in one recent month. The median (25th, 75th) intake of low fat or fat-free dairy products, yogurt, and lactose-reduced dairy products were 0.0 (0.0, 107.1) g/day, 0.0 (0.0, 0.0) g/day and 0.0 (0.0, 0.0) g/day, respectively. As shown in Table 3, those who had a better dairy knowledge level had a significantly higher intake of general dairy products and low-fat or fat-free dairy products. Compared with participants who had no LI-related symptoms, the general dairy product intake amount was significantly lower in participants with symptoms; however, the lowest intake amount was shown in the “unsure” group. There were no significant intake differences in low fat or fat-free dairy products, lactose-reduced dairy products, and yogurt among the LI symptoms group, the non-LI symptoms group, and the unsure group.

The linear regression model in Table 4 illustrates that, after adjusting for gender, increment score levels were associated with 36.3 mg/day increase of calcium intake. However, after also adjusting for education and family income, this association did not exist. There was no significant association between protein intake and dairy knowledge level.

## 4. Discussion

Dairy products are considered key to a good quality diet and cannot be replaced by any other foods or supplements [11]. There are more than three thousand years of history of dairy intake in China. However, insufficient intake of dairy products is common in the Chinese population and has a series of consequences including osteoporosis, obesity, diabetes, and other chronic diseases [4,5,9]. In this study, dairy knowledge, attitude, and related practice were investigated in China. The results extended previous research findings by demonstrating that dairy knowledge gaps and inappropriate perceptions exist in Chinese urban adults and impact on their practice.

### 4.1. Knowledge of Dairy Products

According to the KAP model, knowledge is the psychological foundation of behavior. Regrettably, both in developed and developing countries, there are many people lacking nutritional knowledge [20,21]. Although an Iranian study reported an acceptable knowledge level of dairy products, only 52.9 percent of subjects were aware of dairy products and 72.7 percent of subjects knew the health roles of dairy products [16]. For the Chinese population, Sohwan Son et al. reported lower nutritional knowledge scores in Chinese schoolgirls compared with schoolgirls in Korea [22]. In addition, some studies in China found poor nutritional knowledge even among medical students [23]. According to the results of this study, the dairy knowledge of urban Chinese adults is poor, especially with respect to food security and newly updated knowledge aspects, such as LI. Weak nutritional knowledge has been reported to be associated with a series of inappropriate dietary behaviors [17]. In this survey, we also found that participants with poor dairy knowledge were more likely to have a lower dairy product intake and a lower chance of selecting low-fat or fat-free milk. Furthermore, poor knowledge resulted in an insufficient intake of calcium. Fortunately, as a result of nutritional education, dietary behaviors can change [17]. An Iranian study found that after informing students about the variety of dairy products (including yogurt, milk, and ice cream) and the benefits of their consumption, there was a decrease in perceived barriers to dietary calcium and an increase in milk and dairy produce consumption [24]. These findings indicated that nutritional education is highly necessary in the Chinese population, especially for males, those with a lower education level, and those in a poor economic state. Meanwhile, dairy knowledge should be regularly updated and provided to the target recipients.

### 4.2. Attitude toward Dairy Products

It is also worth noting that, although educational intervention improves nutritional knowledge, there is often a big gap between knowledge and practice, and attitude always plays a crucial role in nutritional behaviors [21,25]. In this study, we found that over half of the participants could not correctly perceive that they had a nutritional problem due to insufficient intake of dairy products. The insufficient intake of dairy products was actually the top nutritional problem among the studied population.

According to previous studies, people with LI always demonstrate lower intake of dairy products. In this study, only 15.2 percent of participants reported having LI-related symptoms, which was far lower than the estimated LI prevalence in China [12]. We found that those who were unsure whether they had symptoms or not had the lowest intake of dairy products. In addition, only 21.7 percent of participants with symptoms were aware that these symptoms might be associated with LI. For those LI people, there are many flexible choices of dairy product, such as lactose-reduced milk and yogurt, that are all easily accessible on the Chinese market [26]. In contrast, there were no differences in intake of lactose-reduced milk and yogurt among participants with different perceptions. The intake rate of lactose-reduced milk and yogurt was extremely low in this study. Based on these results, we inferred that lack of perception of dairy intolerance might be an important factor contributing to the low intake of dairy products, and perception bias might greatly hinder the effective selection of appropriate dairy products.

## 5. Conclusions

In conclusion, knowledge gaps surrounding dairy products and self-perception bias exist in Chinese urban adults, which impact the quantity and quality of dairy intake. Since this study only recruited an urban population, knowledge of and attitude toward dairy products in the rural population remains unknown and is expected to be worse. According to the Chinese nutritional problem of insufficient intake of dairy products, nutritional intervention should be conducted (1) to enhance dairy knowledge, especially regarding LI; (2) to change the perception bias of dairy products; and (3) to educate people in the effective selection of appropriate dairy products that suit their health status.

## Figures and Tables

**Table 1:** The social-demographic characteristics of participants.

		N	%
**City level**	First-tier cities	654	38.2
	Non–first tier cities	1060	61.8
**Gender**	Male	582	34.0
	Female	1132	66.0
**Age**	18–44 years	634	37.0
	45–64 years	578	33.7
	≥65 years	502	29.3
**Education**	Junior high school or below	565	33.1
	Senior high school	704	41.2
	Bachelor’s degree or above	439	25.7
**Family monthly income** (RMB: yuan)	<3000	361	21.2
	3000–4999	489	28.7
	5000–7999	367	21.5
	≥8000	488	28.6
**Family monthly expenditure on food** (RMB: yuan)	<1000	511	29.9
	1000–2999	880	51.4
	≥3000	320	18.8
**Average personal monthly expenditure on dairy products** (RMB: yuan)	<10	423	24.7
	11–50	479	28.0
	>100	810	47.3

**Table 2:** Ordinal logistic regression for socio-demographic indicators of dairy knowledge in Chinese urban adults (*n* = 1714).

Indicator Variable		ORs	95% CI	*p*
**City level**	First-tier cities	−0.04	−0.25, 0.16	0.691
	Non–first tier cities			
**Gender**	Male	−0.24	−0.44, −0.05	0.016
	Female			
**Age**	18–44 years	0.18	−0.08, 0.45	0.169
	45–64 years	0.19	−0.04, 0.42	0.111
	≥65 years			
**Education**	Junior high school or below	−1.20	−1.50, −0.90	<0.001
	Senior high school	−0.36	−0.61, −0.13	0.004
	Bachelor’s degree or above			
**Family monthly income** (RMB: yuan)	<3000	−0.35	−0.66, −0.05	0.024
	3000–4999	−0.13	−0.40, 0.13	0.328
	5000–7999	−0.17	−0.44, 0.10	0.229
	≥8000			
**Family monthly expenditure on food** (RMB: yuan)	<1000	−0.34	−0.65, −0.03	0.033
	1000–2999	−0.00	−0.26, 0.27	0.975
	≥3000			
**Average personal monthly expenditure on dairy products** (RMB: yuan)	<10	−0.35	−0.59, −0.20	0.006
	11–50	0.03	−0.24, 0.30	0.827
	>100			

A chi-squared test was performed prior to the regression and all the socio-demographic indicators were involved in the regression model. The dairy knowledge used in the model was categorized for three groups as −4~0, 1~3 and ≥4 points and assigned a value of 1, 2, and 3 respectively.

**Table 3:** Dairy intake of Chinese urban adults with different dairy knowledge levels and different self-perceptions of lactose intolerance (LI).

Dairy Product and Nutrients Intake		Score of Dairy Knowledge			*p*	Self-Reception of LI-Related Symptoms			*p*
		−4 to 0	1 to 3	4 to 6		Yes	No	Unsure	
		N = 478	N = 695	N = 523		N = 260	N = 1289	N = 165	
Total dairy products (g/day)	Mean	77.8	118.5	136.5		78.5	125.5	66.1	
	Median (25th, 75th)	8.6 (0.0, 107.1)	85.7 (0.0, 200.0)	107.1 (25.0, 220.0)	<0.001	21.4 (0.0, 107.1)	96.4 (8.3, 220.0)	0.0 (0.0, 0.0)	<0.001
Low fat and fat-free dairy products (g/day)	Mean	13.9	16.2	20.0		22.3	15.5	19.9	
	Median (25th, 75th)	0.0 (0.0, 0.0)	0.0 (0.0, 0.0)	0.0 (0.0, 0.0)	0.002	0.0 (0.0, 0.0)	0.0 (0.0, 0.0)	0.0 (0.0, 0.0)	0.146
Yogurt intake (g/day)	Mean	13.9	16.2	20.0		28.8	27.5	16.5	
	Median (25th, 75th)	0.0 (0.0, 0.0)	0.0 (0.0, 0.0)	0.0 (0.0, 0.0)	0.595	0.0 (0.0, 0.0)	0.0 (0.0, 0.0)	0.0 (0.0, 0.0)	0.771
Lactose-reduced dairy products (g/day)	Mean	1.9	3.0	1.3		2.6	2.3	0.6	
	Median (25th, 75th)	0.0 (0.0, 0.0)	0.0 (0.0, 0.0)	0.0 (0.0, 0.0)	0.595	0.0 (0.0, 0.0)	0.0 (0.0, 0.0)	0.0 (0.0, 0.0)	0.671

Nonparametric test was used to examine the intake differences among subjects with different knowledge levels and different self-perception of LI-related symptoms.

**Table 4:** Linear regression models on knowledge levels of calcium and protein intake among Chinese urban adults (*n* = 1714).

Predictors	Unstandardized Coefficient	95% (CI)	t	*p*
Calcium (mg/day)				
Model 1 ^a^	36.3	(5.3, 67.3)	2.294	0.022
Model 2 ^b^	25.6	(−5.8, 57.0)	1.598	0.110
Protein (g/day)				
Model 1 ^a^	1.84	(−0.07, 3.76)	1.886	0.059
Model 2 ^b^	1.45	(−0.49, 3.40)	1.467	0.147

The independent factor dairy knowledge used in this model was categorized for three groups as −4~0, 1~3 and ≥4 points and assigned a value of 1, 2, and 3 respectively. ^a^ Model 1 adjusted for gender; ^b^ Model 2 adjusted for gender education level and family income level.
